# 新型冠状病毒肺炎流行期肺部手术的初步建议

**DOI:** 10.3779/j.issn.1009-3419.2020.03.01

**Published:** 2020-03-20

**Authors:** 昕 李, 明辉 刘, 青春 赵, 仁旺 刘, 洪兵 张, 明 董, 嵩 徐, 洪林 赵, 森 韦, 作庆 宋, 钢 陈, 军 陈

**Affiliations:** 300052 天津，天津医科大学总医院肺部肿瘤外科 Department of Lung Cancer Surgery, Tianjin Medical University General Hospital, Tianjin 300052, China

**Keywords:** 新型冠状病毒肺炎, 肺占位性病变, 外科手术治疗, 2019 novel coronavirus disease (COVID-19), Lung occupying lesions, Surgical treatment

## Abstract

2019年12月，中国确诊首例新型冠状病毒肺炎患者，疫情发展对中国乃至世界产生了巨大影响。对于肺部发现占位性病变患者的整体诊疗流程也因疫情而不能常规开展。对于胸外科医生而言，外科手术的介入时机需要慎重选择。全国的胸外科同道应根据疫情的改变及对新型冠状病毒肺炎认识的不断更新共同制定诊治流程和路径。在此，我们仅依据自己的认识提出初步建议，以供参考和进一步讨论。

## Abstract (en)

In December 2019, China diagnosed the first patient with 2019 novel coronavirus disease (COVID-19), and the following development of the epidemic had a huge impact on China and the whole world. For patients with lung occupying lesions, the whole process of diagnosis and treatment can not be carried out as usual due to the epidemic. For thoracic surgeons, the timing of surgical intervention should be very carefully considered. All thoracic surgeons in China should work together to develop the proper procedures for the diagnosis and treatment in this special situation, and continuously update the recommendations based on epidemic changes and further understanding of COVID-19. Here, we only offer some preliminary suggestions based on our own knowledge for further reference and discussion.

自2019年12月以来，席卷整个中国并在全球多个国家出现的新型冠状病毒肺炎（novel coronavirus pneumonia，NCP，简称新冠肺炎）引起了全球重视，2020年2月12日，世界卫生组织（World Health Organization, WHO）将这一病毒导致的疾病正式命名为COVID-19。整个疫情给中国医疗、政治、经济领域均带来了巨大的影响。新冠肺炎病原体为新型冠状病毒，初称为2019新冠状病毒（2019-nCoV），随后国际病毒分类委员会统一命名为SARS-CoV-2。目前认为该病毒属于β属冠状病毒，有包膜，颗粒呈圆形或椭圆形，常为多形性，直径60 nm-140 nm。此病毒具有可人传人且传染性强的特征，主要经呼吸道飞沫和接触传播，气溶胶和消化道等传播途径尚待明确。潜伏期约1 d-14 d，多为3 d-7 d，以发热、乏力、干咳为主要临床表现，少数患者伴有鼻塞、流涕、咽痛和腹泻等症状。重症患者多在发病1周后出现呼吸困难和/或低氧血症，严重者快速进展为急性呼吸窘迫综合征、脓毒症休克、难以纠正的代谢性酸中毒和出/凝血功能障碍。值得注意的是重症、危重症患者病程中可表现为中低热，甚至无明显发热。实验室检查发现，发病早期外周血白细胞总数正常或降低，淋巴细胞计数减少，部分患者可出现肝酶、乳酸脱氢酶（lactate dehydrogenase, LDH）、肌酶和肌红蛋白增高；部分危重者可见肌钙蛋白增高。多数患者C反应蛋白（C reactive protein, CRP）和血沉升高，降钙素原正常。严重者D-二聚体升高、外周血淋巴细胞进行性减少。在鼻咽拭子、痰、下呼吸道分泌物、血液、粪便等标本中可检测出新型冠状病毒核酸。多数患者预后良好，少数患者病情危重，老年人和有慢性基础疾病者预后较差，儿童病例症状相对较轻^[1]^。

根据国家整体防控方案要求，绝大部分地区启动了限制收支、限制交通、社区封闭式管理等措施，以减少病毒扩散。在这种严峻形势下，对肺部发现占位性病变患者的诊断治疗流程必然会造成一定的影响。部分良性病变因生长较慢可以暂缓外科手术，但肺部恶性病变的患者，延误治疗时机可能会造成肿瘤进展和转移，影响患者预后，甚至危及患者生命。规范治理、简化流程，为全国的胸外科同道共同制定诊治流程和路径建言献策，同时应根据疫情的改变及对疾病的进一步认识更新该建议。我们在此仅提出初步建议，以供参考和讨论。

1.鼓励发现肺部占位性病变的患者就近就诊、当地治疗，尽量减少患者及家属的流动带来的交叉感染。

2.伴有发热、咳嗽、喘憋等呼吸道症状的肺部占位性病变患者应在当地政府指定发热门诊首诊，若为疑似感染患者则收入指定隔离病房，待排除感染，且胸部计算机断层扫描（computed tomography, CT）明确肺部存在占位性病变后可转入胸外科继续诊治。

3.经常规检查，如正电子发射计算机断层显像（positron emission tomography-CT, PET-CT）或经皮肺穿刺活检提示为良性病变，若需要择期手术治疗的患者，建议患者院外随诊观察（不短于3个月），待疫情结束或相对稳定后择期手术治疗。

4.经常规检查，如PET-CT或经皮肺穿刺活检提示为恶性病变，若肿瘤为中心型（疫情防控期间不建议接受支气管镜检查），可建议患者先接受新辅助治疗，待疫情结束或相对稳定后择期手术治疗。

5.中心型恶性病变伴随大量咯血（特殊时期不拘泥于咳血量，应结合患者一般情况确定），或累及主气道伴随严重呼吸困难、病情危重的患者，经科室讨论，从技术角度评估可完整切除病灶的患者，可予急诊手术治疗。

6.对于PET-CT或经皮肺穿刺活检考虑为恶性病变的外周型实性结节，直径≤3 cm，疫情防控期间建议短期定期随诊（每月1次），若随访期内结节增大超过20%，应考虑手术治疗。结节直径≥3 cm，应考虑手术治疗。

7.肺部磨玻璃密度结节（ground-glass nodules, GGNs）：全国肺部筛查试验的研究结果显示：对于4 mm-5 mm的GGNs每年复查胸部CT的恶性风险为0.4%；6 mm-7 mm、8 mm-14 mm、15 mm-19 mm每6个月复查CT的恶性风险分别为1.1%、3.0%、5.2%；结节直径≥20 mm的每3个月复查胸部CT的恶性风险为10.9%^[2]^。因此我们建议，疫情防控期间，不论是纯GGNs，还是混合性GGNs，或是多发性GGNs（多发性GGNs需排除新冠病毒感染），均建议以随诊复查为主，不建议开展外科手术；对于直径5 mm以下的，不短于1年复查；直径6 mm-19 mm的，6个月后复查胸部CT；直径≥20 mm的，不短于3个月复查胸部CT^[3]^，以减少感染风险。同时，亦应高度重视，由于新冠肺炎肺部影像学特征是肺部磨玻璃样改变，对于肺部新发现的磨玻璃样结节或占位，均应该有至少3个月以上的随诊，以排除新冠肺炎的肺部改变。

总体上，我们可将常规肺部手术分为两类：（1）择期手术：定义为可观察3个月以上的手术，包括直径 < 3 cm的各种GGNs、70%以上概率的良性可能性结节或占位，能够保守治疗的肺部良性疾病，如支气管扩张伴咯血等，此类手术在疫情期间建议不做或慎做；（2）限期手术：定义为1个月内最好开展的手术，包括诊断明确的肺癌患者，或者直径 > 3 cm且临床诊断考虑为肺部恶性肿瘤的患者，此类手术应该在完全排除新冠肺炎的基础上进行。各类急诊手术不在此讨论范畴。

在疫情不稳定期间，如需开展胸外科常规手术，术前需积极排查新冠病毒感染可能，对于CT影像学表现疑似感染或患者出现发热、咳嗽、肌肉酸痛、乏力、咳痰、头痛、腹泻任何症状之一的患者，需按照疑似病例进行处理，可行新冠病毒检测排除感染且在单人病房隔离14 d后予手术治疗，手术前一日建议再次复查血常规、CRP，有条件者复查胸部平扫CT，确定手术是否可安全进行。术中需加强隔离保护措施，防止交叉感染的出现。对于胸外科急诊手术患者，如出现上述症状，在来不及排除新冠病毒潜伏感染的情况下，术中要做好隔离防护措施，同时进行新冠病毒检测，待检测结果回报后决定患者术后去向，疑似感染患者进入隔离病房治疗，阴性患者回普通病房治疗。

术后围术期，建议予患者每日2次监测体温，术后第1天抽血检测CRP、血常规、肝功能、肾功能，每3日复查1次，同时术后常规复查胸部CT。如遇到发热（> 37.3 ℃）情况，先给予对症处理，同时急查CRP及血常规，排除新冠病毒感染后，继续常规治疗。如若48 h发热情况无好转，且除外术后常规发热因素，如积液、手术部位常见病原体感染等，应再次排除新冠病毒感染可能性，并给予相应治疗。尽量缩短术后患者住院时间，避免交叉感染。

在我们的建议中没有把新冠病毒的核酸检测作为新冠肺炎阳性与否的金标准，主要原因如下：①虽然目前新冠病毒的核酸检测技术较多，但都没有经过验证，缺乏统一标准，特异性和敏感性不定，特别是敏感性不详；②临床实践中，部分患者早期核酸检测存在一定比例的假阴性，特别是有部分患者早期反复核酸检测为阴性而最终确诊为阳性感染者。故流行病接触史、临床特征、常规含CRP的生化检测和胸部CT的综合为肺外科手术术前的重要判断依据，限期手术术前2周的隔离观察有助于进一步排除新冠肺炎的可能。最后，希望我们的建议能起到抛砖引玉的作用，使更多的胸外科医生参与讨论，形成具有一定共识的规范，既可保障患者的利益，又可减少医务人员的感染^[4]^。
